# Lipopolysaccharides of *Fusobacterium nucleatum* and *Porphyromonas gingivalis* increase RANKL-expressing neutrophils in air pouches of mice

**DOI:** 10.1186/s42826-020-00080-y

**Published:** 2021-01-06

**Authors:** Ae Ri Kim, Yun Kyong Lim, Joong-Ki Kook, Eun-Jung Bak, Yun-Jung Yoo

**Affiliations:** 1 Department of Oral Biology, College of Dentistry, Yonsei University, Seoul, Republic of Korea; 2 Department of Applied Life Science, The Graduate School, Yonsei University, Seoul, Republic of Korea; 3 BK21 PLUS Project, College of Dentistry, Yonsei University, Seoul, Republic of Korea; 4 Korean Collection for Oral Microbiology and Department of Oral Biochemistry, School of Dentistry, Chosun University, Gwangju, Republic of Korea

**Keywords:** Periodontitis, Neutrophils, RANKL, Periodontopathogen

## Abstract

Increases of neutrophils and osteoclasts are pathological changes of periodontitis. RANKL is an osteoclast differentiation factor. The effect of periodontopathogen LPS on RANKL-expressing neutrophils has not been clarified yet. We evaluated numerical changes of RANKL-expressing neutrophils in air pouches of mice injected with LPSs of *Fusobacterium nucleatum* and *Porphyromonas gingivalis.* Mice with air pouches were assigned into saline (C)-, *E. coli* LPS- (Ec LPS)-, *F. nucleatum* LPS (Fn LPS)-, *P. gingivalis* LPS (Pg LPS)-, and Fn LPS and Pg LPS (Fn + Pg LPS)-injected groups. CD11b^+^Ly6G^+^ neutrophils and CD11b^+^Ly6G^+^RANKL^+^ neutrophils in blood and air pouch exudates were determined by flow cytometry. In blood, compared to the C group, the Fn LPS group showed increases of CD11b^+^Ly6G^+^ neutrophils and CD11b^+^Ly6G^+^RANKL^+^ neutrophils whereas the Pg LPS group showed no significant differences. These increases in the Fn LPS group were not different to those in the Ec LPS group. In exudates, Fn LPS and Pg LPS groups showed increases of CD11b^+^Ly6G^+^ neutrophils and CD11b^+^Ly6G^+^RANKL^+^ neutrophils compared to the C group. Increased levels in the Fn LPS group were not different to those in the Ec LPS group, but Pg LPS group was lower than those in the Ec LPS group. In blood and exudates, the Fn + Pg LPS group showed no difference in levels of these neutrophils compared to the Ec LPS group. LPSs of *F. nucleatum* and *P. gingivalis* increased RANKL-expressing neutrophils although the degrees of increases were different. These suggest that periodontopathogen LPS can act as a stimulant to increase RANKL-expressing neutrophils.

## Introduction

Periodontitis is an inflammatory disease caused by several bacteria. Bacteria in subgingival biofilm was grouped into five complexes (red, orange, green, yellow, and purple complexes) by cluster analysis and community ordination technique [1]. Green, yellow, and purple complexes are initial colonizers of the biofilm [2]. *Fusobacterium nucleatum* is one member of the orange complex. *F. nucleatum* bridges initial and later bacterial colonizers in biofilm formation [3–5]. It has virulence factors that can damage host tissue or adhere to host tissue or other bacteria [5]. *Porphyromonas gingivalis* is one member of the red complex that shows close relation with pocket depth and bleeding on probing [1]. *P. gingivalis* is considered to be a keystone pathogen that causes abnormalities in immune response [5, 6].

Lipopolysaccharide (LPS), endotoxin of Gram-negative bacteria, is composed of lipid A, core oligosaccharide, and O-specific polysaccharide. Detailed structures of LPS vary depending on bacterial species and, in some bacterial species, the structures depend on the environment [7–9]. The structural variations of LPS can affect its activities [10]. Both *F. nucleatum* and *P. gingivalis* have LPS as a virulence factor. *F. nucleatum* LPS (Fn LPS) is a stronger stimulator for the secretion of IL-1β and TNF-α than *P. gingivalis* LPS (Pg LPS) in neutrophils, suggesting that secretion levels of cytokines induced by LPSs from various periodontopathogens might play important roles in the onset and progression of periodontal disease [11]. Receptor activator of NF-κB ligand (RANKL) is an osteoclast formation-inducing cytokine associated with alveolar bone resorption in periodontal diseases [12, 13]. It has been reported that RANKL expression is upregulated in osteoblasts after Pg LPS treatment [14]. In addition, it has been suggested that RANKL-expressing neutrophils are involved in osteoclast formation in chronic obstructive pulmonary disease and rheumatoid arthritis [15, 16]. However, the effect of periodontopathogen LPS on RANKL-expressing neutrophils has not been determined yet.

Neutrophils are representative inflammatory cells that express cytokines. Neutrophils derived from stem cells of bone marrow can infiltrate into infection site through circulation [17, 18]. A previous in vivo study has shown that RANKL-expressing neutrophils are increased in air pouches of mice injected with *Escherichia coli* LPS (Ec LPS) [19]. Air pouch generated on the back of a mouse has been used as an in vivo model to estimate infiltration of neutrophils into stimulant-injected air pouch [20]. To determine whether periodontopathogen LPS could act as a stimulant to increase of RANKL-expressing neutrophils, RANKL-expressing neutrophils in Pg LPS and Fn LPS-injected air pouches of mice were estimated in the present study.

## Methods/experimental

### Air pouch induction in mouse dorsum

Eight weeks male C57BL/6 mice (Orient Bio) were kept in polypropylene cages under specific pathogen-free conditions with temperature of 22 °C, humidity of 60%, and a 12 h/12 h of light/dark cycle. The mice were fed standard chow and provided with water ad libitum. Protocols for animal experiment were approved by the Institutional Animal Care and Use Committee of Yonsei University (Approval Number: 2017–0109 and 2018–0147).

**Fig. 1:**
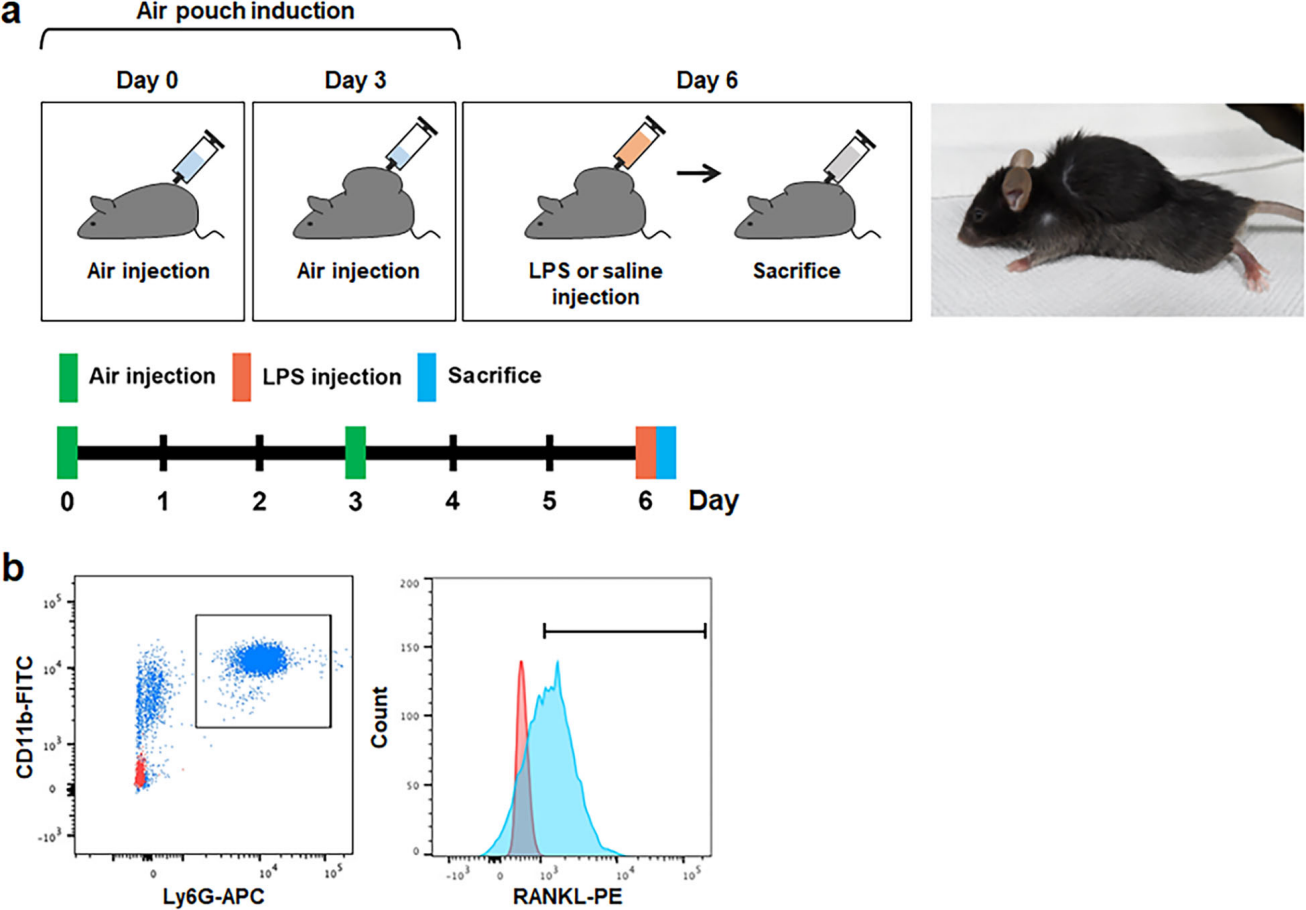
Schematic diagram showing the experiment with air pouches of mice. **a** Air pouch generation. Sterilized air was injected subcutaneously into the dorsum of each mouse on day 0 and day 3. At six days after the initial air injection, mice received either LPS (Ec LPS, Fn LPS or Pg LPS) or saline injection into air pouches, and sacrificed after 6 h. **b** Gating pictures for CD11b^+^Ly6G^+^ neutrophils and CD11b^+^Ly6G^+^RANKL^+^ neutrophils in exudates of air pouches of mice. CD11b^+^Ly6G^+^ neutrophils were analyzed in live cells gated based on SSC (granularity) and FSC (relative size). Numbers of CD11b^+^Ly6G^+^RANKL^+^ neutrophils were then analyzed. The red dot or region represents unstained sample of Ec LPS group and the blue dot or region represents stained sample of Ec LPS group. LPS, lipopolysaccharide

To generate air pouch, 5 ml of sterilized air were injected subcutaneously into the dorsum of each mouse on day 0 and then 3 ml of additional sterile air was reinjected on day 3 (Fig. 1a) [20]. Air injections were performed under anesthesia with a mixture of Zoletil 50 (30 mg/kg; Virbac, Carros, France) and Rompun (10 mg/kg; Bayer Korea).

### LPS injection into air pouch

Ec LPS (Sigma-Aldrich, St Louis, Mo, USA) was used as a positive control. Fn LPS (Korean Collection for Oral Microbiology, Gwangju, Korea) and Pg LPS (Invivogen, San Diego, CA, USA) were used as periodontopathogen LPS. Three individual experiments with 3 different groups were conducted. First experiment was conducted to saline-administered control group (C, *n* = 7), Ec LPS-administered alone group (Ec LPS, *n* = 5), and Fn LPS-administered alone group (Fn LPS, *n* = 8). Second experiment was conducted to C group (*n* = 6), Ec LPS group (*n* = 5), and Pg LPS-administered alone group (Pg LPS, *n* = 8). Third experiment was conducted to C group (*n* = 5), Ec LPS group (*n* = 8), and Fn LPS and Pg LPS-administered combination group (Fn + Pg LPS, *n* = 7). In the LPS alone experiment, the concentration of LPS in Ec LPS, Fn LPS or Pg LPS group was 2 μg/ml. In the combination experiment, the concentration of LPS was 2 μg/ml per bacterium in the Fn + Pg LPS group and 4 μg/ml in the Ec LPS group. Mice were administered LPS or saline at 6 days after the initial air injection and then were sacrificed 6 h later.

### Flow cytometry

Immediately after sacrifice, blood sampled through cardiac perforation was transferred into ethylene diamine tetra acetic acid (EDTA) tubes. Agranulocytes and granulocytes were separated from blood using two density gradients of Histopaque-1083 and -1119 (Sigma-Aldrich) [21]. Briefly, 2 ml of Histopaque-1083 was carefully placed on 3 ml of Histopaque-1119. PBS and blood in the same volume were mixed, placed on Histopaque-1083, and centrifuged at 750 × *g* for 30 min at room temperature. The upper layer and the lower layer of Histopaque-1083 were mixed with PBS and centrifuged at 700 × *g* for 5 min at 4 °C.

To collect exudates from air pouches, 2 ml of 2 mM EDTA was injected into air pouch of each mouse. After collected exudates were centrifuged at 300 × g for 5 min at 4 °C, cells were fixed with 2% paraformaldehyde and counted with a hemocytometer. Collected cells were incubated with anti-CD16/32 antibody (Ab) for 30 min at 4 °C to block non-specific reactions in which anti-Ly6G and anti-RANKL Abs bind to the Fc receptor on the cell membrane. CD11b is an adhesion molecule of neutrophils that can bind to ligands on endothelial cells [22]. Ly6G, a glycosylphosphatidylinositol-linked protein, is a specific marker that distinguish neutrophils from other leukocytes [23]. The cells were incubated with FITC-labeled anti-CD11b Ab, APC-labeled anti-Ly6G Ab, and PE-labeled anti-RANKL Ab (1:100 dilutions per 10^6^ cells; BioLegend, San Diego, CA, USA) for 1 h at 4 °C. CD11b^+^Ly6G^+^ neutrophils and CD11b^+^Ly6G^+^RANKL^+^ neutrophils were analyzed by flow cytometry (BD Fortessa, Becton & Dickinson, Franklin Lakes, NJ, USA). Gating was performed using BD FACSDiva™ software (Becton & Dickinson, Franklin Lakes, NJ, USA) (Fig. 1b). First, live cells were gated based on scale scatter (SSC, granularity) and forward scatter (FSC, relative size). Then CD11b^+^Ly6G^+^ neutrophils were analyzed. Finally, CD11b^+^Ly6G^+^RANKL^+^ neutrophils were analyzed. Unstained cells were used for gating of negative control.

### Statistical analysis

The statistical significance of data was determined with the Kruskal–Wallis test (*p* < 0.05), a nonparametric test. If the differences were significant, Man-Whitney U test (*p* < 0.017) was additionally performed. Data are expressed as mean ± standard error (SE). All statistical analyses were performed using SPSS (IBM SPSS Statistics version 25, Armonk, NY, USA).

## Results

### Effects of Fn LPS on RANKL-expressing neutrophils in blood and exudates of air pouches

**Fig. 2:**
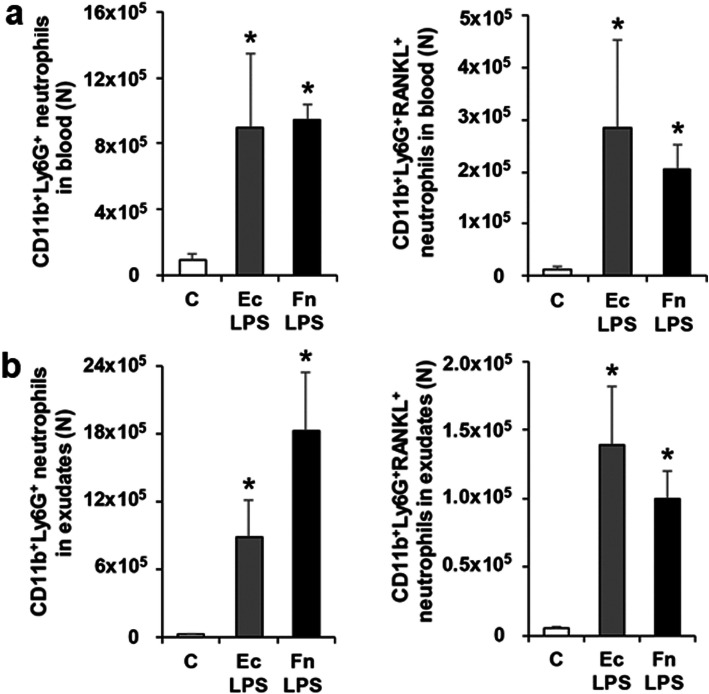
Neutrophils and RANKL-expressing neutrophils in mice with Fn LPS-injected air pouches. **a** Numbers of CD11b^+^Ly6G^+^ neutrophils and CD11b^+^Ly6G^+^RANKL^+^ neutrophils in blood. **b** Numbers of CD11b^+^Ly6G^+^ neutrophils and CD11b^+^Ly6G^+^RANKL^+^ neutrophils in exudates. Data are presented as mean ± SE. *p < 0.017 vs C. C, control; Ec, *Escherichia coli*; Fn, *Fusobacterium nucleatum*; LPS, lipopolysaccharide; N, number

In order to evaluate the effect of Fn LPS on numbers of RANKL-expressing neutrophils in blood and exudates of air pouches of mice, numbers of CD11b^+^Ly6G^+^ neutrophils and CD11b^+^Ly6G^+^RANKL^+^ neutrophils were measured by flow cytometry (Fig. 2a and b). In blood and exudates, numbers of neutrophils and RANKL-expressing neutrophils in the Ec LPS group were higher than these in the C group. In the Fn LPS group, numbers of neutrophils and RANKL-expressing neutrophils were not significantly different to the Ec LPS group. These suggest that Fn LPS can increase neutrophils and RANKL-expressing neutrophils in blood and exudates of air pouches.

### Effects of Pg LPS on RANKL-expressing neutrophils in blood and exudates of air pouches

**Fig. 3:**
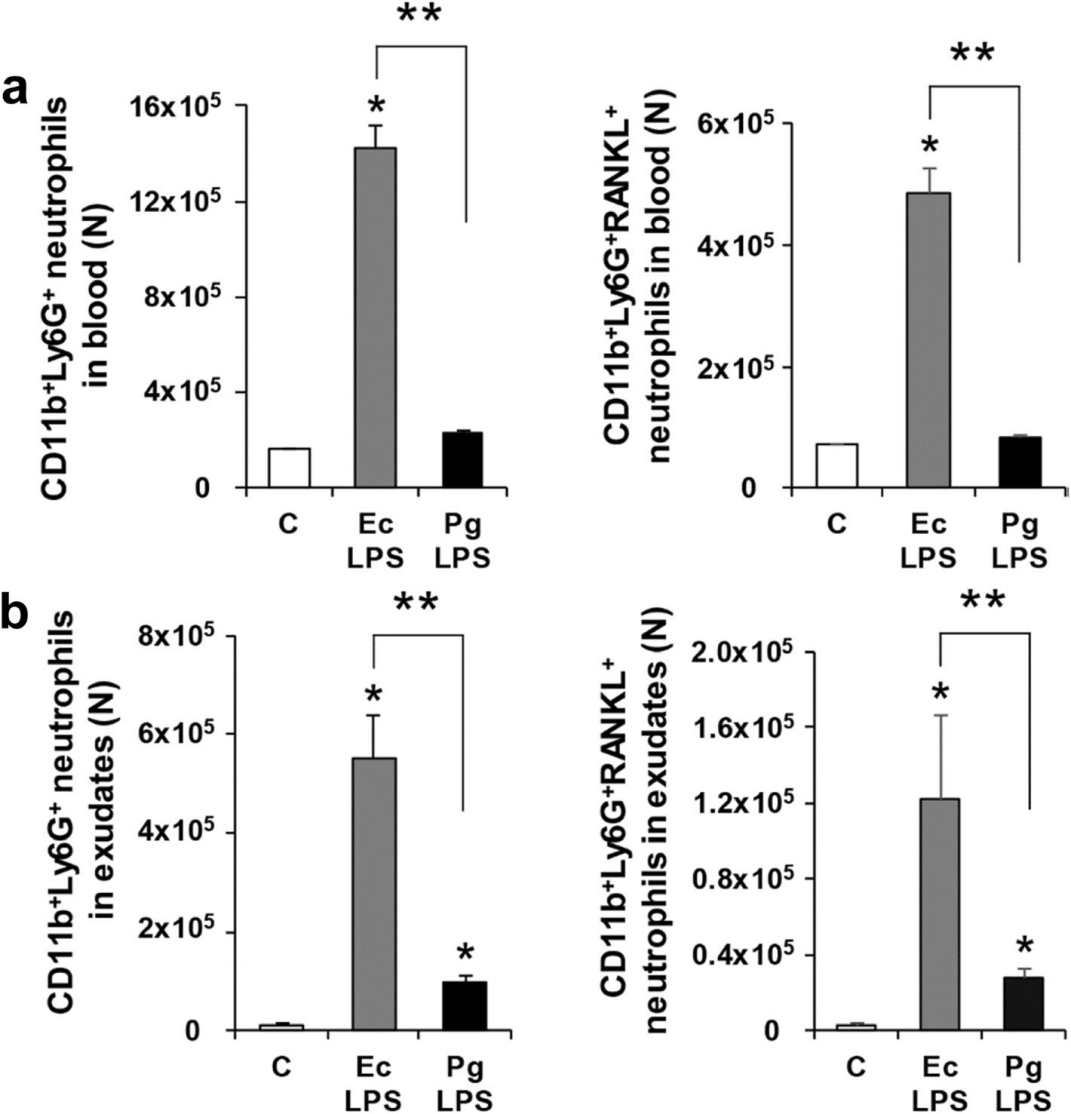
Neutrophils and RANKL-expressing neutrophils in mice with Pg LPS-injected air pouches. **a** Numbers of CD11b^+^Ly6G^+^ neutrophils and CD11b^+^Ly6G^+^RANKL^+^ neutrophils in blood. **b** Numbers of CD11b^+^Ly6G^+^ neutrophils and CD11b^+^Ly6G^+^RANKL^+^ neutrophils in exudates. Data are presented as mean ± SE. *p < 0.017 vs C. **p < 0.017. C, control; Ec, *Escherichia coli*; Pg, *Porphyromonas gingivalis*; LPS, lipopolysaccharide; N, number

In order to evaluate the effect of Pg LPS on numbers of RANKL-expressing neutrophils in blood and exudates of air pouches, numbers of neutrophils and RANKL^+^ neutrophils were measured by flow cytometry (Fig. 3a and b). In blood of the Pg LPS group, numbers of neutrophils and RANKL-expressing neutrophils were significantly lower than those in the Ec LPS group, but were not significantly different to the C group (Fig. 3a). In exudates of the Pg LPS group, numbers of neutrophils and RANKL-expressing neutrophils were higher than those in the C group, but significantly lower than those in the Ec LPS group (Fig. 3b). These suggest that Pg LPS can increase neutrophils and RANKL-expressing neutrophils in exudates of air pouches, but not in blood.

### Effects of Fn LPS and Pg LPS combination on RANKL-expressing neutrophils in blood and exudates of air pouches

**Fig. 4:**
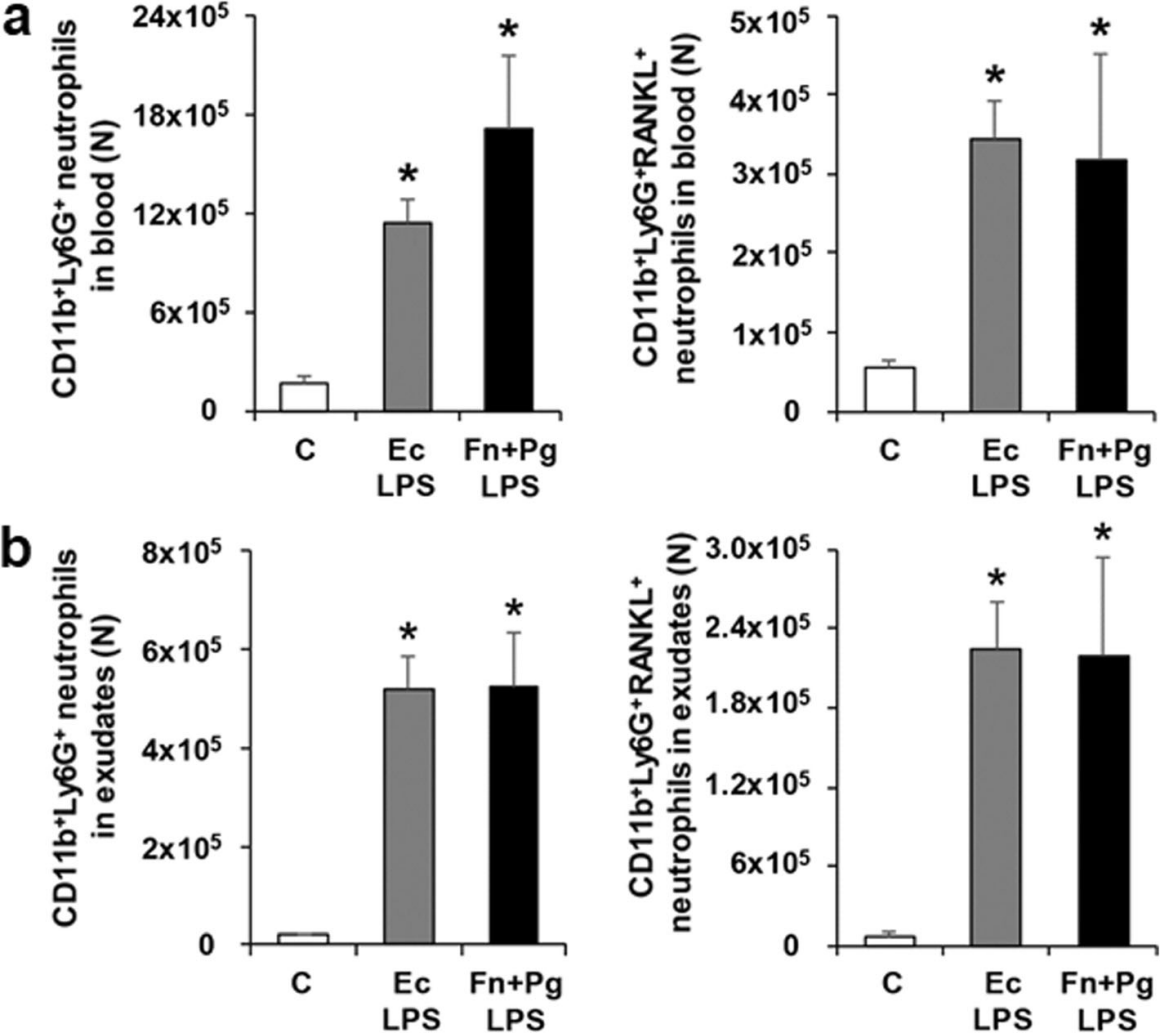
Neutrophils and RANKL-expressing neutrophils in mice with Fn LPS and Pg LPS-injected air pouches. **a** Numbers of CD11b^+^Ly6G^+^ neutrophils and CD11b^+^Ly6G^+^RANKL^+^ neutrophils in blood. **b** Numbers of CD11b^+^Ly6G^+^ neutrophils and CD11b^+^Ly6G^+^RANKL^+^ neutrophils in exudates. Data are presented as mean ± SE. *p < 0.017 vs C. C, control; Ec, *Escherichia coli*; Fn, *Fusobacterium nucleatum;* Pg, *Porphyromonas gingivalis*; LPS, lipopolysaccharide; N, number

To evaluate the effect of co-administration of Fn LPS and Pg LPS on numbers of neutrophils and RANKL-expressing neutrophils, Fn LPS and Pg LPS in combination were injected into air pouches of mice (Fig. 4a and b). In both blood and exudates, numbers of neutrophils and RANKL-expressing neutrophils in the Fn + Pg LPS group were increased, were not significantly different to those in the Ec LPS group. These suggest that co-administration of Fn LPS and Pg LPS can increase neutrophils and RANKL-expressing neutrophils in blood and exudates of air pouches similar to Ec LPS.

## Discussion

Identification of neutrophil response to periodontopathogens is necessary to understand the pathogenesis of periodontitis. This study showed that LPSs from *F. nucleatum* and *P. gingivalis* could increase RANKL-expressing neutrophils in air pouches of mice.

Lipid A of LPS is composed of disaccharide and acyl chains and its compositions are known to differ according to bacteria [7]. After Ec LPS injection, blood and air pouch exudates showed increased levels of RANKL-expressing neutrophils, similar to results of other air pouch study using neutrophil maker Ly6B [19]. These findings indicate that Ec LPS can increase RANKL-expressing neutrophils. Fn LPS has been reported to have a hexa-acylated lipid A structure similar to Ec LPS [5, 10]. In the present study, increases of RANKL-expressing neutrophils in blood and exudates after Fn LPS injection were not different to those after Ec LPS injection. This might be related to the structural similarity between Fn LPS and Ec LPS.

In the case of *P. gingivalis*, lipid A exhibits heterogeneous acylation patterns (penta-acylated and tetra-acylated lipid A) depending on environmental conditions [9]. *P. gingivalis* exposed to low hemin conditions forms penta-acylated lipid A. On the other hand, its exposure to high hemin conditions forms tetra-acylated lipid A. Pg LPS with tetra-acylated lipid A different from the lipid A structure of Ec LPS was used in this study. In gingival epithelial cells, Ec LPS up-regulates expression of β-defensin, an antimicrobial molecule, but Pg tetra-acylated LPS down-regulates its expression, suggesting that different lipid A structure could differentially modulate host immune response [24]. In another study, the level of TNF-α induced by Pg LPS in neutrophils was less than that induced by Ec LPS [25]. In the present study, Pg LPS increased RANKL-expressing neutrophils less than Ec LPS. Thus, effects of Pg LPS and Ec LPS on RANKL-expressing neutrophils were different. A previous study has shown that Fn LPS can stimulate more IL-1β secretion in neutrophils than Pg LPS [11]. These suggest that neutrophil responses to Fn LPS injection and Pg LPS injection might be different. Taken together, LPS from *F. nucleatum* and *P. gingivalis* can act as stimulants to increase RANKL-expressing neutrophils, although their abilities might be different.

Periodontitis is a polymicrobial infectious disease [1, 26]. Thus, we administered Fn LPS and Pg LPS simultaneously to air pouches of mice. Numbers of RANKL-expressing neutrophils in blood and exudates were increased in the group treated with a combination of Fn LPS and Pg LPS, similar to those in the group treated with Ec LPS. Our results confirmed that RANKL-expressing neutrophils were increased in the presence of two bacterial LPSs, suggesting that RANKL-expressing neutrophils could affect the progression of periodontitis. RANKL is an essential factor of osteoclast differentiation [13]. Increases of RANKL-expressing neutrophils under inflammatory conditions such as presence of periodontopathogen LPS in air porches suggest that RANKL-expressing neutrophils might be associated with osteoclast formation during periodontitis. An in vivo study is needed in the future to confirm the relationship between osteoclast formation and RANKL-expressing neutrophils during periodontitis.

## Conclusions

These findings propose that periodontopathogen LPS act as a stimulant to increase RANKL-expressing neutrophils. This study is meaningful in that it reports the effect of periodontopathogen LPS on RANKL-expressing neutrophils.

## Data Availability

All the data generated or analyzed during this study are included in this published article.

## Abbreviations

Ab: Antibody
C: Control
Ec: *Escherichia coli*
EDTA: Ethylene diamine tetra acetic acid
Fn: *Fusobacterium nucleatum*
LPS: Lipopolysaccharide
N: Number
Pg: *Porphyromonas gingivalis*
RANKL: Receptor activator of NF-κB ligand
SE: Standard error

## References

[1] Socransky SS, Haffajee AD, Cugini MA, Smith C, Kent RL (1998). Microbial complexes in subgingival plaque. J Clin Periodontol.

[2] Socransky SS, Haffajee AD (2000). Dental biofilms: difficult therapeutic targets. Periodontol.

[3] Bradshaw DJ, Marsh PD, Watson GK, Allison C. Role of *Fusobacterium nucleatum* and coaggregation in anaerobe survival in planktonic and biofilm oral microbial communities during aeration. Infect Immun. 1998;66(10):4729–32. 10.1128/iai.66.10.4729-4732.1998 PMC108582 9746571

[4] Kolenbrander PE, Andersen RN, Moore LV (1989). Coaggregation of *Fusobacterium nucleatum*, *Selenomonas flueggei*, *Selenomonas infelix*, *Selenomonas noxia*, and *Selenomonas sputigena* with strains from 11 genera of oral bacteria. Infect Immun.

[5] de Andrade KQ, Almeida-da-Silva CLC, Coutinho-Silva R. Immunological pathways triggered by* Porphyromonas gingivalis* and *Fusobacterium nucleatum*: therapeutic possibilities? Mediators Inflamm. 2019;2019:7241312. 10.1155/2019/7241312 PMC6612971 31341421

[6] Hajishengallis G, Darveau RP, Curtis MA (2012). The keystone-pathogen hypothesis. Nat Rev Microbiol.

[7] Wang X, Quinn PJ. Lipopolysaccharide: biosynthetic pathway and structure modification. Prog Lipid Res. 2010;49(2):97–107. 10.1016/j.plipres.2009.06.002 19815028

[8] Wilkinson SG (1996). Bacterial lipopolysaccharides-themes and variations. Prog Lipid Res.

[9] Al-Qutub MN, Braham PH, Karimi-Naser LM, Liu X, Genco CA, Darveau RP (2006). Hemin-dependent modulation of the lipid A structure of *Porphyromonas gingivalis* lipopolysaccharide. Infect Immun.

[10] Asai Y, Makimura Y, Kawabata A, Ogawa T (2007). Soluble CD14 discriminates slight structural differences between lipid as that lead to distinct host cell activation. J Immunol.

[11] Yoshimura A, Hara Y, Kaneko T, Kato I (1997). Secretion of IL-1β, TNF-α, IL-8 and IL-1ra by human polymorphonuclear leukocytes in response to lipopolysaccharides from periodontopathic bacteria. J Periodontal Res.

[12] Cochran DL (2008). Inflammation and bone loss in periodontal disease. J Periodontol.

[13] Takahashi N, Udagawa N, Suda T (1999). A new member of tumor necrosis factor ligand family, ODF/OPGL/TRANCE/RANKL, regulates osteoclast differentiation and function. Biochem Biophys Res Commun.

[14] Kassem A, Henning P, Lundberg P, Souza PP, Lindholm C, Lerner UH. *Porphyromonas gingivalis* stimulates bone resorption by enhancing RANKL (receptor activator of NF-κB ligand) through activation of Toll-like receptor 2 in osteoblasts. J Biol Chem. 2015;290(33):20147–58. 10.1074/jbc.M115.655787 PMC4536425 26085099

[15] Hu X, Sun Y, Xu W, Lin T, Zeng H (2017). Expression of RANKL by peripheral neutrophils and its association with bone mineral density in COPD. Respirology.

[16] Poubelle PE, Chakravarti A, Fernandes MJ, Doiron K, Marceau AA (2007). Differential expression of RANK, RANK-L, and osteoprotegerin by synovial fluid neutrophils from patients with rheumatoid arthritis and by healthy human blood neutrophils. Arthritis Res Ther.

[17] Hajishengallis E, Hajishengallis G (2014). Neutrophil homeostasis and periodontal health in children and adults. J Dent Res.

[18] Silva LM, Brenchley L, Moutsopoulos NM (2019). Primary immunodeficiencies reveal the essential role of tissue neutrophils in periodontitis. Immunol Rev.

[19] Chakravarti A, Raquil MA, Tessier P, Poubelle PE (2009). Surface RANKL of Toll-like receptor 4-stimulated human neutrophils activates osteoclastic bone resorption. Blood.

[20] Vandal K, Rouleau P, Boivin A, Ryckman C, Talbot M, Tessier PA (2003). Blockade of S100A8 and S100A9 suppresses neutrophil migration in response to lipopolysaccharide. J Immunol.

[21] Swamydas M, Luo Y, Dorf ME, Lionakis MS. Isolation of mouse neutrophils. Curr Protoc Immunol. 2015;110:3.20.1–15. 10.1002/0471142735.im0320s110 PMC4574512 26237011

[22] Parkos CA (1997). Molecular events in neutrophil transepithelial migration. BioEssays.

[23] Lee PY, Wang JX, Parisini E, Dascher CC, Nigrovic PA (2013). Ly6 family proteins in neutrophil biology. J Leukoc Biol.

[24] Lu Q, Darveau RP, Samaranayake LP, Wang CY, Jin L (2009). Differential modulation of human β-defensins expression in human gingival epithelia by *Porphyromonas gingivalis* lipopolysaccharide with tetra- and penta-acylated lipid A structures. Innate Immun.

[25] Gu JY, Liu YJ, Zhu XQ, Qiu JY, Sun Y (2020). Effects of endotoxin tolerance induced by *Porphyromonas gingivalis* lipopolysaccharide on inflammatory responses in neutrophils. Inflammation.

[26] Darveau RP (2010). Periodontitis: a polymicrobial disruption of host homeostasis. Nat Rev Microbiol.

