# Correction to: Lipopolysaccharides of Fusobacterium nucleatum and Porphyromonas gingivalis increase RANKL-expressing neutrophils in air pouches of mice

**DOI:** 10.1186/s42826-021-00099-9

**Published:** 2021-08-09

**Authors:** Ae Ri Kim, Yun Kyong Lim, Joong-Ki Kook, Eun-Jung Bak, Yun-Jung Yoo

**Affiliations:** 1 Department of Oral Biology, College of Dentistry, Yonsei University, Seoul, Republic of Korea; 2 Department of Applied Life Science, The Graduate School, Yonsei University, Seoul, Republic of Korea; 3 BK21 PLUS Project, College of Dentistry, Yonsei University, Seoul, Republic of Korea; 4 Korean Collection for Oral Microbiology and Department of Oral Biochemistry, School of Dentistry, Chosun University, Gwangju, Republic of Korea

**Correction to: Lab Anim Res 37, 5 (2021)**

**https://doi.org/10.1186/s42826-020-00080-y**

It was highlighted that in the original article [1] the superscript of the y-axis unit in the right panel of Fig. 3, panel a, was missing (10 should be 10^5^). This Correction article shows the correct Fig. 3. The original article has been updated.

**Fig. 3:**
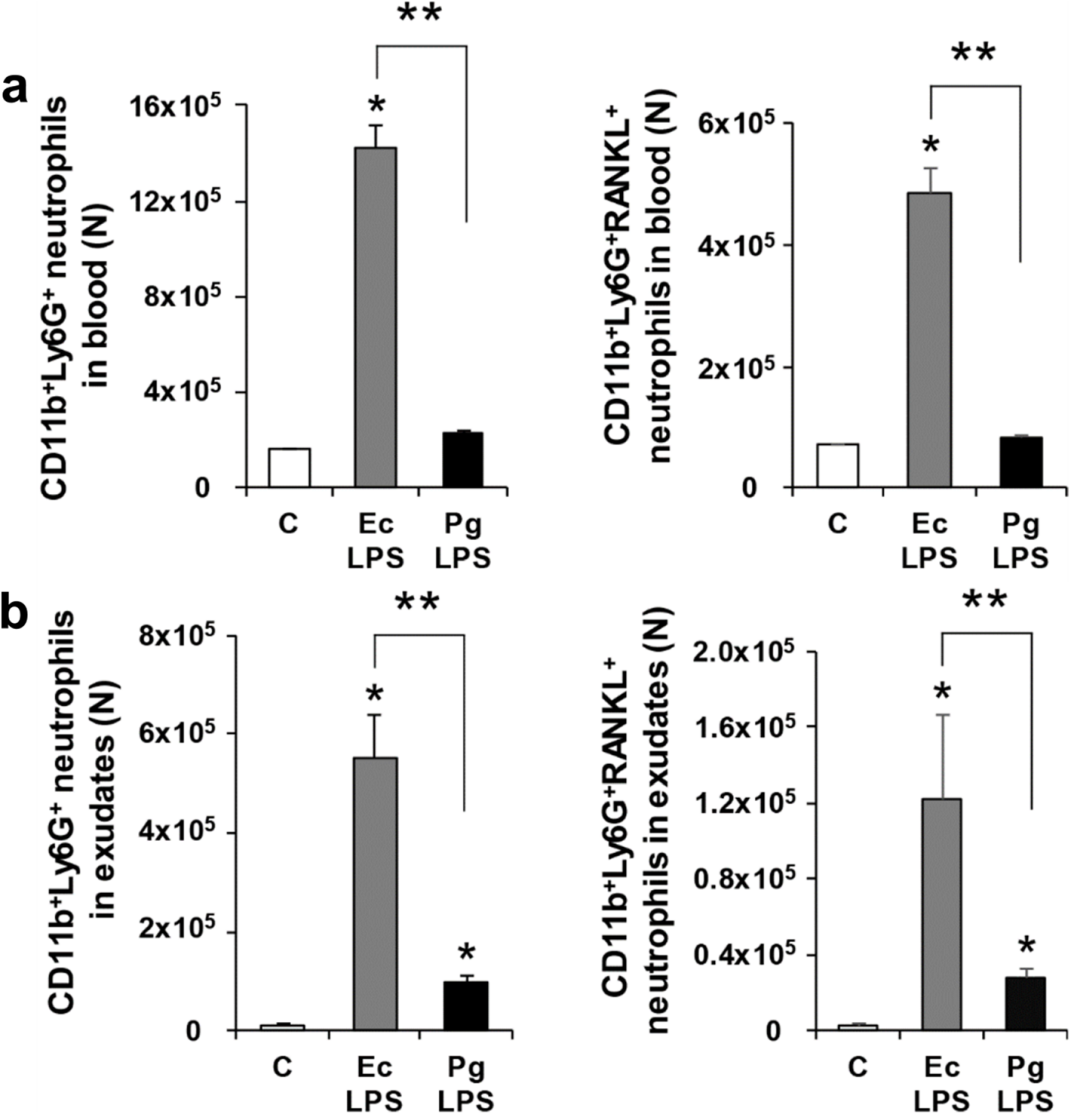
Neutrophils and RANKL-expressing neutrophils in mice with Pg LPS-injected air pouches. **a** Numbers of CD11b^+^Ly6G^+^ neutrophils and CD11b^+^Ly6G^+^RANKL^+^ neutrophils in blood. **b** Numbers of CD11b^+^Ly6G^+^ neutrophils and CD11b^+^Ly6G^+^RANKL^+^ neutrophils in exudates. Data are presented as mean ± SE. *p < 0.017 vs C. **p < 0.017. C, control; Ec, *Escherichia coli*; Pg, *Porphyromonas gingivalis*; LPS, lipopolysaccharide; N, number
